# Concurrent ependymal and ganglionic differentiation in a subset of supratentorial neuroepithelial tumors with *EWSR1-PLAGL1* rearrangement

**DOI:** 10.1186/s40478-024-01809-9

**Published:** 2024-09-03

**Authors:** Julieann C. Lee, Selene C. Koo, Larissa V. Furtado, Alex Breuer, Mohammad K. Eldomery, Asim K. Bag, Pat Stow, Gary Rose, Trisha Larkin, Rick Sances, Bette K. Kleinschmidt-DeMasters, Jenna L. Bodmer, Nicholas Willard, Murat Gokden, Sonika Dahiya, Kaleigh Roberts, Kelsey C. Bertrand, Daniel C. Moreira, Giles W. Robinson, Jun Qin Mo, David W. Ellison, Brent A. Orr

**Affiliations:** 1 Department of Pathology, Neuropathology, St. Jude Children’s Research Hospital, 262 Danny Thomas Place, Memphis, TN 38105 USA; 2 Department of Pathology, Molecular Pathology, St. Jude Children’s Research Hospital, Memphis, TN USA; 3 Department of Diagnostic Imaging, St. Jude Children’s Research Hospital, Memphis, TN USA; 4 Department of Pathology, Nemours Children’s Hospital, Wilmington, DE USA; 5 Department of Pediatrics, St. Joseph’s Hospital, Tampa, FL USA; 6 Department of Pathology, East TN Children’s Hospital, Knoxville, TN USA; 7 Department of Pathology, University of Colorado, Denver, CO USA; 8 Department of Pathology, University of Arkansas for Medical Sciences, Little Rock, AR USA; 9 Division of Neuropathology, Department of Pathology and Immunology, Washington University, St. Louis, MO USA; 10 Department of Oncology, Division of Neuro-Oncology, St. Jude Children’s Research Hospital, Memphis, TN USA; 11 Department of Pathology, Rady Children’s Hospital, University of California School of Medicine, San Diego, CA USA

**Keywords:** *PLAGL1*, *PLAGL2*, *EWSR1-PLAGL1*, Ganglionic differentiation, Ependymal-like, Neuroepithelial tumor

## Abstract

Neuroepithelial tumors with fusion of *PLAGL1* or amplification of *PLAGL1/PLAGL2* have recently been described often with ependymoma-like or embryonal histology respectively. To further evaluate emerging entities with PLAG-family genetic alterations, the histologic, molecular, clinical, and imaging features are described for 8 clinical cases encountered at St. Jude (*EWSR1-PLAGL1* fusion n = 6; *PLAGL1* amplification n = 1; *PLAGL2* amplification n = 1). A histologic feature observed on initial resection in a subset (4/6) of supratentorial neuroepithelial tumors with *EWSR1-PLAGL1* rearrangement was the presence of concurrent ependymal and ganglionic differentiation. This ranged from prominent clusters of ganglion cells within ependymoma/subependymoma-like areas, to interspersed ganglion cells of low to moderate frequency among otherwise ependymal-like histology, or focal areas with a ganglion cell component. When present, the combination of ependymal-like and ganglionic features within a supratentorial neuroepithelial tumor may raise consideration for an *EWSR1-PLAGL1* fusion, and prompt initiation of appropriate molecular testing such as RNA sequencing and methylation profiling. One of the *EWSR1-PLAGL1* fusion cases showed subclonal INI1 loss in a region containing small clusters of rhabdoid/embryonal cells, and developed a prominent ganglion cell component on recurrence. As such, *EWSR1-PLAGL1* neuroepithelial tumors are a tumor type in which acquired inactivation of *SMARCB1* and development of AT/RT features may occur and lead to clinical progression. In contrast, the *PLAGL2 and PLAGL1* amplified cases showed either embryonal histology or contained an embryonal component with a significant degree of desmin staining, which could also serve to raise consideration for a PLAG entity when present. Continued compilation of associated clinical data and histopathologic findings will be critical for understanding emerging entities with PLAG-family genetic alterations.

**Supplementary Information:**

The online version contains supplementary material available at 10.1186/s40478-024-01809-9.

## Introduction

The three PLAG-family genes are *PLAGL1* (PLAG like 1) located on chromosome 6q24, *PLAG1* located on 8q12, and *PLAGL2* located on 20q11, which encode C2H2 zinc finger transcription factors with involvement in various processes including cell cycle regulation and proliferation [1, 19]. *PLAG1* (pleomorphic adenoma gene 1) rearrangement occurs in pleomorphic adenoma and lipoblastoma, with rearrangement of *PLAGL1* more recently described in a set of supratentorial neuroepithelial tumors with frequent ependymal-like features [15]. Two of the 40 cases in the series by Sievers et al. [15], were considered glioneuronal tumor or anaplastic ganglioglioma on initial diagnosis within the available supplementary details. Other reports on CNS tumors with *EWSR1-PLAGL1* rearrangement [10, 12, 13, 16, 20, 21] have included a frontal lobe glioneuronal tumor with a ganglion cell component [10], a tumor identified by retrospective methylation profiling of anaplastic gangliogliomas [13, 15], and a case diagnosed as an INI1-deficient atypical teratoid/rhabdoid tumor [12]. In contrast, *PLAGL1* or *PLAGL2* amplification has been described in brain tumors composed of, or with areas of, primitive embryonal-like cells lacking robust expression of GFAP and synaptophysin, with desmin staining in a subset [8, 17]. While PLAG amplified cases were initially recognized by methylation profiling evaluation that didn’t match to an established group [4], a small percentage of tumors with methylation profile of “embryonal tumor with PLAG-family amplification” actually lack amplification [8, 18], with *PLAG1* fusion found in the absence of amplification in rare cases [18]. The current study further adds detailed histologic evaluation, associated clinical outcome data, and imaging findings for a small cohort of neuroepithelial tumors with PLAG-family genetic alterations, expanding the histopathologic and clinical spectrum while emphasizing the utility of appearance and staining attributes during the evaluation process.

## Materials and methods

The histologic, molecular, clinical, and imaging features were compiled for a cohort of cases encountered clinically at St. Jude and collaborating institutions, with approval by the institutional review board (IRB) of St. Jude Children’s Research Hospital. All cases underwent methylation profiling and associated large-scale copy number analysis at St. Jude, with subsequent additional evaluation of.idat files on DKFZ classifier version 12.5 through molecularneuropathology.org. Additionally, all cases were evaluated by RNA sequencing at St. Jude. Cases with adequate sample availability (4/8) underwent targeted panel sequencing analysis on the St. Jude Pedi Panel v1.1 which evaluates 362 genes for single nucleotide variants and indels. Alternatively, two cases (2/8) were evaluated by clinical triple platform sequencing (combined whole genome, whole exome, and RNA sequencing) using fresh/frozen tissue collected intraoperatively with concurrent germline sequencing [14]. Sequencing for two cases (2/8) occurred separately on targeted platforms at other academic centers (Children’s Hospital of Colorado or University of California San Diego), with one case also having chromosomal microarray analysis at University of California San Diego. For a more detailed description of molecular testing methods, please see the corresponding supplemental file. Imaging of the tumors was evaluated by a pediatric neuroradiologist. All the anatomic imaging sequences and diffusion weighted imaging was reviewed. The minimum apparent diffusion coefficient was manually calculated using a 0.1 cm^2^ region of interest on the areas of the tumor that visually appeared to have the lowest values, and it was expressed in units of 10–6 mm^2^/s.

## Results

### Clinical and imaging features

Eight neuroepithelial tumors with PLAG-family genetic alterations were encountered at St. Jude (*EWSR1-PLAGL1* fusion n = 6; *PLAGL1* amplification n = 1; *PLAGL2* amplification n = 1), with the clinical and imaging features summarized in Table 1 and Supplementary Table 1. Supratentorial *PLAGL1*-fused cases (4F:2 M) were detected by RNA sequencing with a corresponding methylation profile of neuroepithelial tumor *PLAGL1* fused (DKFZ 12.5, Table 2), ranging in age from 9 months to 14 years at time of initial diagnosis. Amplified cases included a cerebellar mass with *PLAGL2* amplification in a 2-year-old female, and a left temporal mass with *PLAGL1* amplification in a 4-year-old male.

**Table 1:** Clinical and radiographic features of neuroepithelial tumors with PLAG-family genetic alterations

Patient	Age at Dx	Sex	PLAG-alteration	Tumor size and location	Extent of resection	Treatment	Clinical status	Length of follow-up
#1	9 mo	F	*EWSR1-PLAGL1* fusion	8.9 cm left frontal mass	NTR, GTR, GTR	carboplatin/etoposide	Residual/recurrent disease by imaging at 3 months with additional resection at 6 months, additional recurrence/resection at 1.2 years for local rapid growth, alive with no evidence of disease by imaging at 1.5 years	1.5 yrs
#2	11 mo	M	*EWSR1-PLAGL1* fusion	6 cm right frontal mass	GTR	ACNS0334, then modified MEMMAT, followed by tazometostat	Recurrence/resection at 7 months with intracranial multifocal disease, died of disease progression at 11 months	11 months
#3	2 yrs	F	*EWSR1-PLAGL1* fusion	9.1 cm right occipital mass	NTR, GTR, GTR	SJYC07 chemotherapy, continued observation	Recurrence/resection at 1.6 years, additional recurrence/resection at 9 years, stable nodularity at resection cavity by imaging at 10.3 years	10.3 yrs
#4	12 yrs	F	*EWSR1-PLAGL1* fusion	6.6 cm right occipital mass	NTR, GTR	focal proton beam 54 CGE, chemotherapy per ACNS0831 maintenance	Additional resection at 2 months for minimal residual neoplasm, alive with no evidence of disease by imaging at 2 years	2 yrs
#5	14 yrs	M	*EWSR1-PLAGL1* fusion	8 cm right frontal mass, multicystic	Not available	Not available	Not available	Not available
#6	11 yrs	F	*EWSR1-PLAGL1* fusion	6.6 cm left frontal mass	GTR	Focal radiation 54 Gy	Alive with no evidence of disease at 13 months, treatment related imaging changes	13 months
#7	2 yrs	F	*PLAGL2* amplification	5.3 cm right cerebellar mass	GTR	Chemotherapy, autologous bone marrow transplant	Alive with no evidence of disease by imaging at 4.8 years	4.8 yrs
#8	4 yrs	M	*PLAGL1* amplification	7.2 cm left temporal mass	STR, STR, NTR	CSI (23.4CGE) followed by focal boost (54CGE), SJMB12 N1 chemotherapy	Minimal residual disease at 2 months, no definitive disease by imaging at 7 months	7 months

*NTR* near toral resection, *GTR* gross total resection, *STR* subtotal resection, *CSI* craniospinal irradiation

**Fig. 1:**
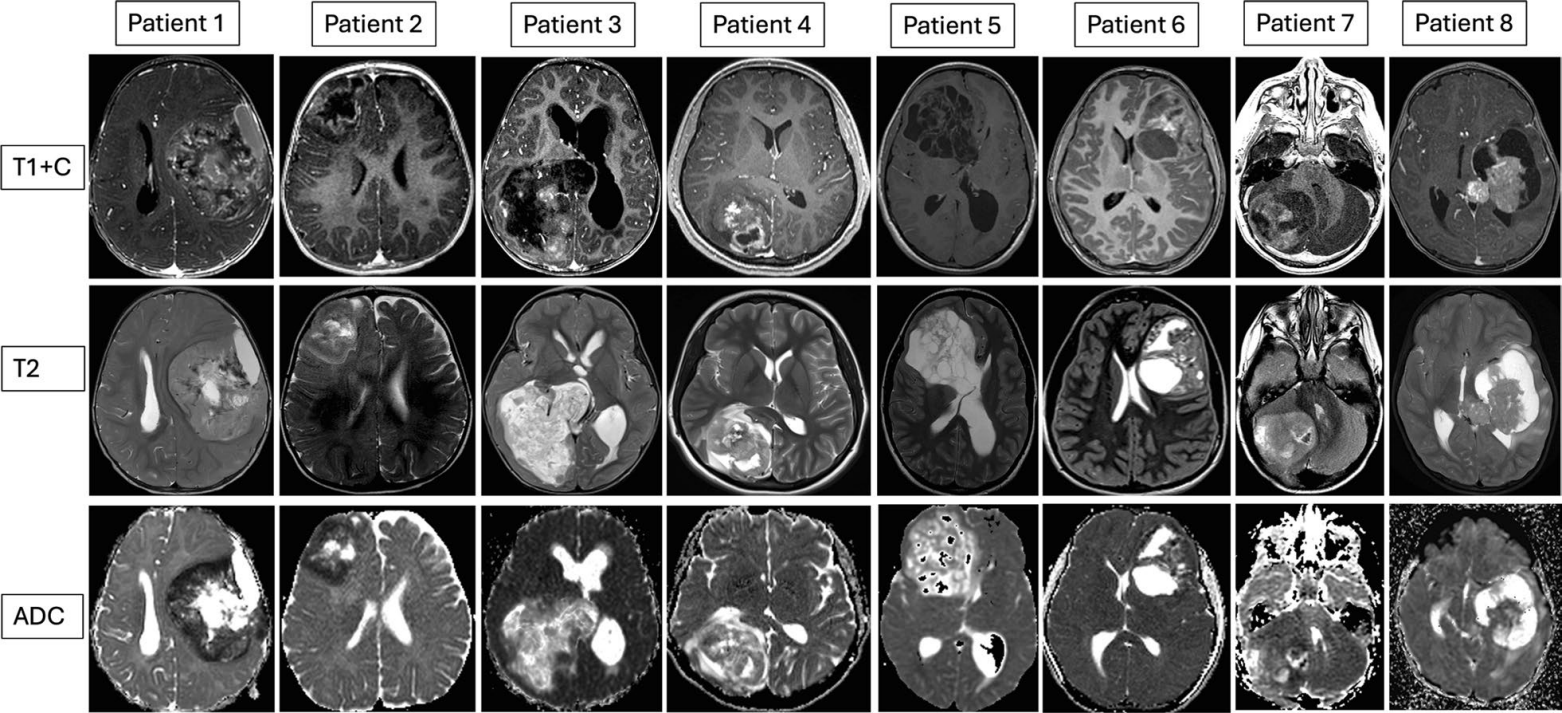
Representative pre-operative brain MRI images of neuroepithelial tumors with PLAG-family genetic alterations are shown. Cases #1–6 have *EWSR1-PLAGL1* rearrangement, while Case #7 and #8 have *PLAGL2* amplification or *PLAGL1* amplification respectively. The top row demonstrates post-contrast T1-weighted sequences, the middle row demonstrates T2-weighted sequences, and the bottom row shows apparent diffusion coefficient maps. Heterogeneous enhancement and diffusion restriction of the solid component are the most common findings, except for tumor #5, which was multicystic and showed no enhancement or diffusion restriction. Tumor cysts were present in case #5, 6, and 8. Additional imaging details are available in supplementary Table 1

All of the PLAG-family tumors in this series were well-defined with involvement of the adjacent dura/meninges, occurring as large (> 5 cm) supratentorial tumors, except for the one tumor with *PLAGL2* amplification located in the cerebellum. The shared imaging findings included heterogeneous enhancement and diffusion restriction of the solid component, except for patient #5 whose tumor was multicystic [Fig. 1]. Hemorrhage was present in 6/8 cases, peritumoral edema was present in 3/8 cases, and a cystic component was present in 3/8 cases. Four of the 8 tumors had associated bone remodeling (either thinning or osteopenia).

### Histologic and immunohistochemical features

The *EWSR1-PLAGL1* cases appeared predominantly as solid glial neoplasms, with areas of infiltration. In a few of the cases clear demarcation from adjacent brain parenchyma could be seen in selected areas. The architecture was either ependymal-like with perivascular anucleate zones (perivascular pseudorosettes), subtly ependymal with less pronounced perivascular zones, or in one case a combination of ependymal-like areas and areas with nuclear clusters reminiscent of an ependymoma/subependymoma.

**Fig. 2:**
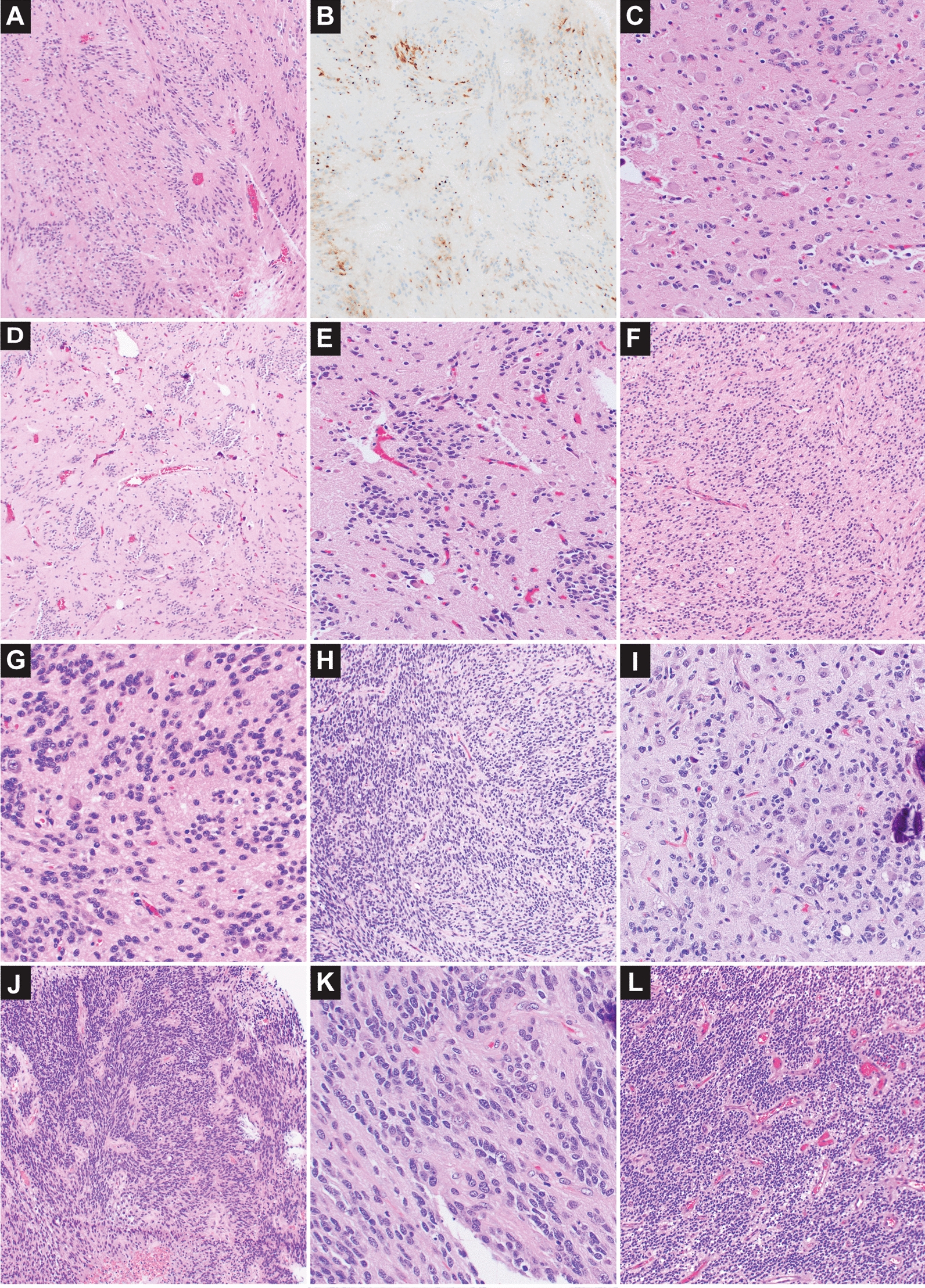
Histology of neuroepithelial tumors with *EWSR1-PLAGL1* rearrangement. Concurrent ependymal and ganglionic differentiation was observed on initial resection in 4/6 cases with *EWSR1-PLAGL1* rearrangement in this series. The most prominent example occurred in a tumor with combined ependymoma/subependymoma-like histology (Case #4) including areas with intermixed clusters of ganglion cells (**A**–**E**). Paranuclear dot-like staining for EMA was only convincingly present in Case #4 (**B**). Case #5 also showed lower-grade histologic features and had interspersed dysmorphic ganglion cells of low to moderate frequency among otherwise subtly ependymal-like histology; the ganglion cells were small in size (**F**–**G**). Case #3 contained ganglion cells in regions of lower-cellularity with occasional microcalcifications, the adjacent areas with greater ependymal quality showed smaller sized cells and variable cellularity (**H**–**I**). Case #6 was predominantly ependymal-like with focal areas containing ganglionic cells, there was regionally increased cellularity (**J**–**K**). Case #1 lacked a ganglion cell component on initial resection, and showed higher-grade histologic features with increased cellularity (**L**), elevated Ki-67 labeling and mitotic index, as well as areas of necrosis

While there was not an appreciable ganglion cell component on initial resection for case #1, which was cellular with perivascular anucleate zones [Fig. 2L], occasional dysmorphic ganglion cells were seen within the tumor on the recurrences. The degree of ganglion cell involvement on initial resection varied [Fig. 2, Table 2] from prominent clusters of ganglion cells within ependymoma/subependymoma-like areas [Case #4, Fig. 2A–E], ganglion cells in in areas of lower-cellularity with adjacent areas of increased cellularity and smaller cells with ependymal features [Case #3, Fig. 2H–I], to interspersed ganglion cells of low to moderate frequency among otherwise subtle ependymal-like histology [Case #5, Fig. 2F–G], and focal areas containing ganglion cells within an ependymal-like neoplasm [Case #6, Fig. 2J–K].

**Table 2:** Molecular and histologic features of neuroepithelial tumors with PLAG-family genetic alterations

Patient	Methylation profiling (DKFZ 12.5)	RNA sequencing	Copy number profile	DNA sequencing mutations	Histologic appearance	Mitotic index	Ki-67 LI	Necrosis	MVP	IHC
#1	Neuroepithelial tumour, PLAGL1-fused, 0.99	*EWSR1-PLAGL1* fusion	Focal loss on 6q	None detected	1st: regionally ependymal-like, cellular 2nd: ependymal-like with occasional dysmorphic appearing ganglionic cells 3rd: regionally ependymal-like with foci of ganglionic cells	1st: 12/10 hpf	~ 40–60%	Multifocal nonpalisading necrosis	No	GFAP: positive in a subset, areas of absent staining OLIG2: positive in a smaller subset of neoplastic cells EMA: negative Synaptophysin: patchy positivity within the neoplasm p53: nuclear positivity in the vast majority of neoplastic cells
#2	Neuroepithelial tumour, PLAGL1-fused, 0.99	*EWSR1-PLAGL1* fusion	Focal gains/losses on 6q loss of 22q with focal interstitial gain	*SMARCB1* p.R53* 34% allelic frequency	1st: large ependymal-like areas, subclonal INI1 loss in region with small clusters of rhabdoid/embryonal cells 2nd: leptomeningeal disease with frequent ganglion cells and INI1 loss	1st: 19/10 hpf in ependymal area 22/10 hpf in area of INI1 loss	~ 40%	Present in first resection nonpalisading	Yes	GFAP: patchy positivity within ependymal-like areas OLIG2: nuclear positivity in a smaller subset of neoplastic cells EMA: rare/focal paranuclear dot-like staining Neurofilament: supports a solid growth pattern Synaptophysin: patchy positivity within ependymal-like areas L1CAM: patchy weak staining Desmin: positive in an intermediate percentage of cells/processes in INI1 retained area INI1: subclonal loss in a region containing small rhabdoid/embryonal clusters
#3	Neuroepithelial tumour, PLAGL1-fused, 0.99	*EWSR1-PLAGL1* fusion	Focal loss on 6q	None detected	1st, 2nd: concurrent ependymal and ganglionic features 3rd: ependymal-like with focal ganglion cells	Variable in first resection, low in subsequent resections 1st: 8/10 hpf in cellular area, low in areas of decreased cellularity 3rd: 3/10 hpf	Variable in first resection, low in subsequent resections 1st: 15–25% in cellular areas, 4–8% in less cellular areas 3rd: predominantly 4–8%, focally up to 15%	Focally present in first resection nonpalisading	No	GFAP: variably positive within the neoplasm OLIG2/SOX10: negative EMA: rare/focal paranuclear dot-like staining Neurofilament: solid and infiltrative pattern in first resection, predominantly solid subsequently Synaptophysin/NeuN: highlights ganglionic cells L1CAM: patchy positivity Desmin: rare filamentous staining
#4	Neuroepithelial tumour, PLAGL1-fused, 0.99	*EWSR1-PLAGL1* fusion	Focal loss on 6q focal loss on 22q	None detected	Concurrent ependymal and ganglionic features	Low	5–8%	No	Small foci	GFAP: positive throughout the neoplasm OLIG2/SOX10: negative EMA: patchy paranuclear dot-like staining Neurofilament: predominantly solid, with areas of infiltration Synaptophysin: highlights areas of ganglionic differentiation L1CAM: negative in majority of the neoplasm, regional positivity Desmin: negative
#5	Neuroepithelial tumour, PLAGL1-fused, 0.99	*EWSR1-PLAGL1* fusion	Focal loss on 6q	None detected	Subtle concurrent ependymal and ganglionic features	Low	8–10%	No	No	GFAP: positive within the neoplasm OLIG2/SOX10: negative EMA: negative Neurofilament: highlights neuronal processes and areas of infiltration Synaptophysin: highlights ganglionic differentiation, and areas of infiltration L1CAM: patchy positivity Desmin: rare positive cells and filaments
#6	Neuroepithelial tumour, PLAGL1-fused, 0.99	*EWSR1-PLAGL1* fusion	Noisy copy number signal on 6q	None detected	Ependymal features with focal areas of ganglionic cells	16/10 hpf	20–30%	Large areas of nonpalisading necrosis	Focal	GFAP: positive in majority of neoplasm, with areas of absent staining OLIG2: negative in vast majority of tumor cells SOX10: negative EMA: essentially negative Neurofilament: supports a solid growth pattern Synaptophysin: no significant staining Desmin: positive in a smaller percentage of neoplastic cells
#7	CNS Embryonal tumour with PLAG-family amplification, 0.99	No recurrent fusion transcripts	*PLAGL2* amplification gain of 2, 3, 7, 8, 11, 12, 19, 21 loss of 10, 22	None detected	Embryonal, vaguely perivascular arrangement	19/10 hpf	~ 35%	Focal	No	GFAP: positive in a smaller subset of neoplastic cells Synaptophysin: focally positive, no significant staining Desmin: positive in a significant percentage of neoplastic cells YAP1: positive GAB1: negative beta-catenin: cytoplasmic
#8	CNS Embryonal tumour with PLAG-family amplification, 0.99	No recurrent fusion transcripts	*PLAGL1* amplification, gain 7, loss 17p	*NCOR2* p.V862fs 16% allelic frequency present in third resection, not detected in second resection	Divergent differentiation with prominent embryonal component, glial elements, and myogenic differentiation	16/10 hpf in primitive component	~ 70% in highest areas, while 8–20% in other areas	Present nonpalisading	Incipient	GFAP: positive regions, with occasional positive cells in other areas Synaptophysin: multiple areas of strong staining, and positive small clusters Desmin: positive in a significant percentage of neoplastic cells Cytokeratin CAM 5.2: focal area with positivity in majority of cells

*1st* first resection, *2nd* second resection, *3rd* third resection, *hpf* high-power field, *LI* labeling index, *MVP* microvascular proliferation, *IHC* immunohistochemistry

**Fig. 3:**
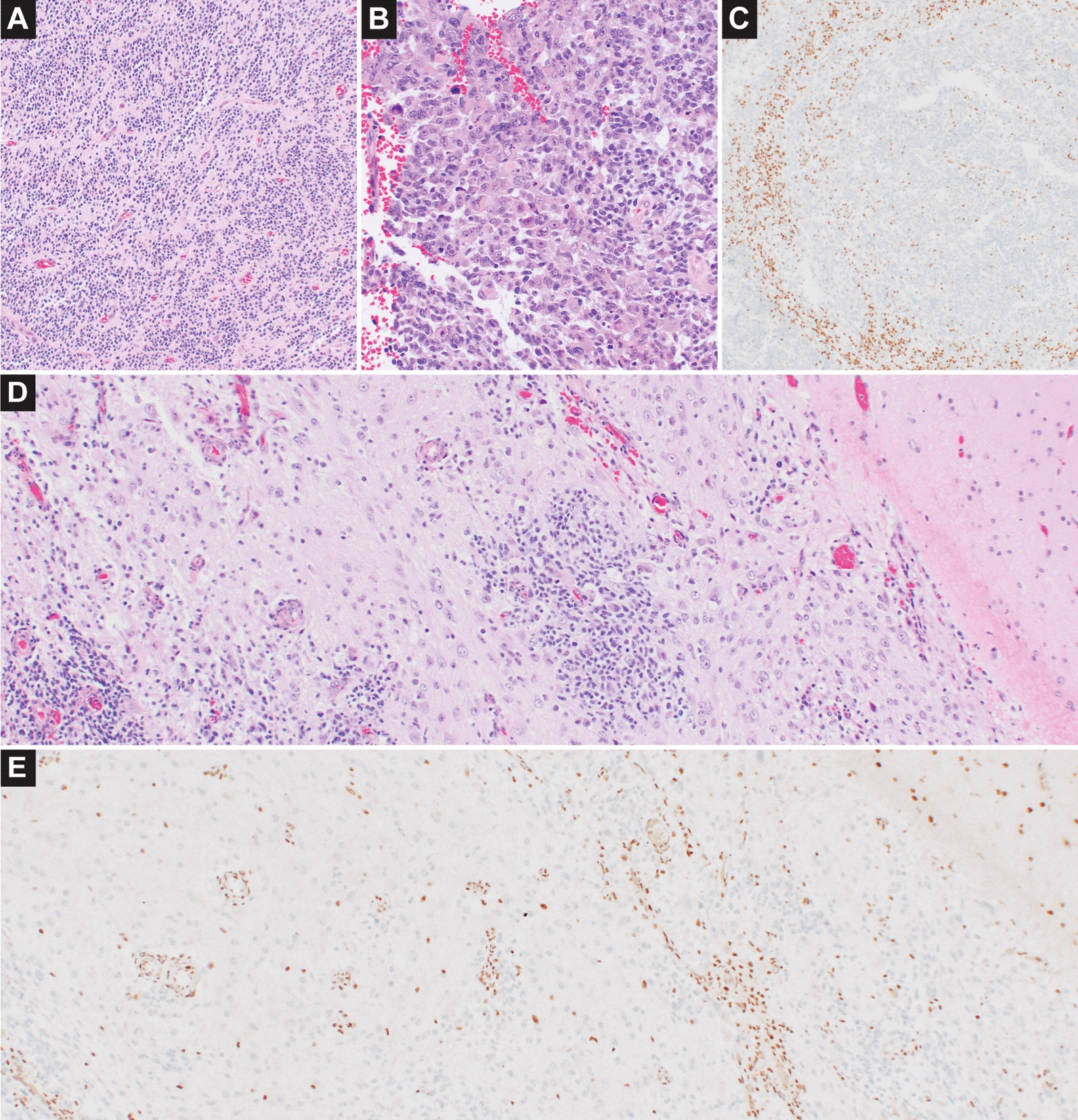
Neuroepithelial tumor with *EWSR1-PLAGL1* fusion and subclonal INI1 loss. On initial resection of case #2 a majority of the neoplasm demonstrated ependymal-like architecture (**A**). However, a selected region of the tumor with increased cellularity contained small clusters of embryonal or rhabdoid cells (**B**) and showed loss of INI1 by immunostaining (**C**). The patient received chemotherapy per ACNS0334 and developed multifocal intracranial disease, with additional resection at 7 months showing dense leptomeningeal involvement including a prominent ganglion cell component (**D**). There was loss of INI1 staining in most neoplastic cells of the second resection, including the small cell component and ganglionic cells (**E**). The patient died of disease progression 11 months after initial resection

Case #2 [Fig. 3A–E] was also a solid cellular predominantly glial appearing neoplasm with ependymal-like architecture [Fig. 3A], but was distinctive in that there was subclonal INI1 loss by immunostaining [Fig. 3C]. The area of INI1 loss involved a more cellular area in which there were small clusters of embryonal/rhabdoid cells [Fig. 3B]. This patient developed multifocal intracranial disease with leptomeningeal involvement. Subsequent resection showed a prominent ganglion cell component at the cortical surface [Fig. 3D], and intermixed smaller cells that were similar in appearance to those of the prior ependymal-like areas but with slightly greater pleomorphism. The vast majority of tumor cells at recurrence, including the ganglion cells and the small cell component, showed INI1 loss [Fig. 3E]. The development of ganglion cells on recurrence in this case may have in-part been treatment related. The region of INI1 inactivation could indicate either ATRT transformation within an ependymal-like neoplasm, or an acquired accompanying mutation associated with tumor progression in an *EWSR1-PLAGL1* neuroepithelial tumor. Methylation profiling separately performed in the region of INI1 loss demonstrated a low subthreshold calibrated score of 0.39 for the methylation class atypical teratoid rhabdoid tumor SHH activated, further supporting a transitional area of the neoplasm with a greater degree of epigenetic homology for AT/RT. The methylation class neuroepithelial tumor *PLAGL1* fused was not represented among the lower calibrated score results in this area of the tumor, though an extremely low score for CNS embryonal tumor with PLAG family amplification was found.

In 2/6 *EWSR1-PLAGL1* fusion cases (#4, #5) mitotic activity was low with infrequent or inconspicuous mitotic figures, which correlated with the absence of necrosis, and lower Ki-67 labeling index (ranging from 5 to 10%). In contrast, 3/6 cases (#1, #2, #6) had an elevated mitotic index with 12, 16, or 19 mitotic figures per 10 high power fields, areas of non-palisading necrosis, and a higher Ki-67 labeling index at or above 30%. While case #3 had variable areas of mitotic activity and Ki-67 on the initial resection and was histologically considered a high-grade neuroepithelial tumor or potentially a type of anaplastic ganglioglioma, the two subsequent resections had lower-grade histologic features. Microvascular proliferation, though present in three cases at least focally, wasn’t a prominent finding in any of the cases. The overall histologic impression when considering cellularity, Ki-67 labeling, mitotic index, and areas of necrosis supports a lower-grade neuroepithelial tumor in 2/6 cases (#4, #5), a higher-grade neuroepithelial tumor in 3/6 cases (#1, #2, #6), and in one case intermixed low and higher-grade areas (#3). The mitotic activity did not specifically correlate with age at diagnosis, as elevated mitotic activity was observed both in patients less than 1 year of age and at 11 years of age.

In *EWSR1-PLAGL1* fusion cases the absence of OLIG2 and SOX10 staining with relatively solid growth pattern are ependymal-like features, though ependymoma type EMA staining was only convincingly present in one case [Fig. 2B] with focal/rare paranuclear dot-like staining in two cases. Neurofilament staining supported a predominantly solid growth pattern in 3/5 cases. There was a mixed solid and infiltrative pattern initially in case #3, with more solid growth in subsequent resections. In case #5 neurofilament and synaptophysin staining suggested both areas of infiltration and a component of neural antigen expression within the tumor. L1CAM did not demonstrate significant strong or diffuse staining within the four cases tested. Desmin staining in two of the higher-grade appearing *PLAGL1* rearranged cases showed positivity in a smaller to intermediate percentage of neoplastic cells and cellular processes.

**Fig. 4:**
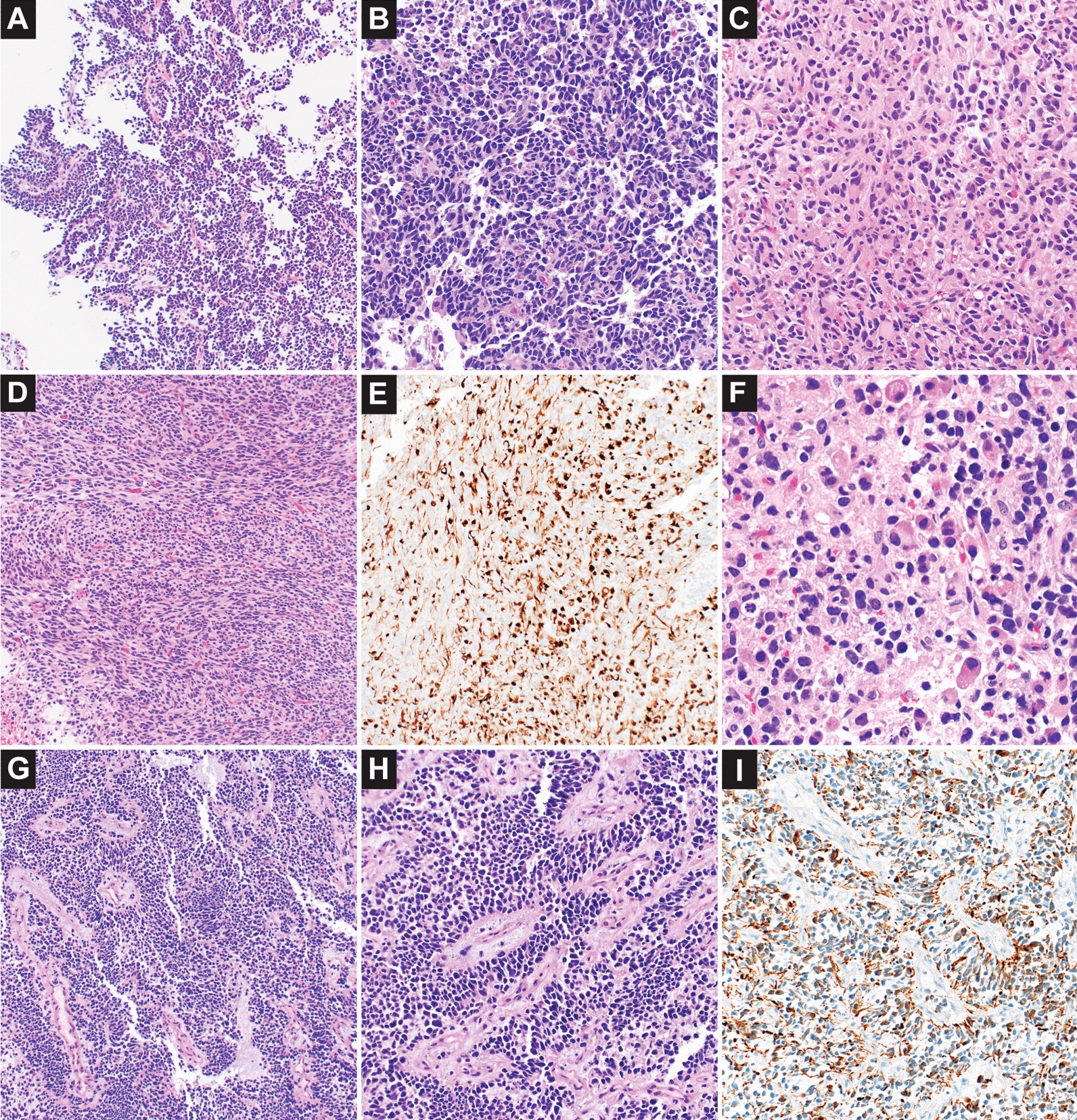
Histology of high-grade neuroepithelial/embryonal tumors with *PLAGL1* or *PLAGL2* amplification. Case #8 (*PLAGL1* amplification) demonstrated divergent differentiation with a prominent embryonal component, glial elements, and myogenic differentiation (**A**–**F**). A loose perivascular/pseudopapillary architecture of primitive cells was seen in selected areas (**A**), with higher magnification showing small hyperchromatic crowded nuclei with nuclear molding (**B**). The histology varied with other areas having a greater degree of eosinophilic cytoplasm and glial fibrillarity (**C**) or spindled cells in fascicular arrangement (**D**). Desmin immunostaining was positive in a significant percentage of neoplastic cells (**E**), with myogenic qualities in rare cells with densely eosinophilic cytoplasm or elongation (**F**). Case #7 (*PLAGL2* amplification) showed primitive cells with a vaguely perivascular arrangement and occasional foci of myxoid material (**G**–**H**); desmin positivity also involved a significant percentage of neoplastic cells (**I**)

While areas of the *PLAGL1* amplified tumor showed strong positivity for GFAP or synaptophysin, sometimes with overlapping areas of staining, other areas of the tumor were largely negative for both lineage markers with small clusters or occasional cells showing positivity. Despite this variation for GFAP and synaptophysin staining, desmin was positive in a significant percentage of neoplastic cells throughout the neoplasm [Fig. 4E], with rare cells showing densely eosinophilic cytoplasm or elongation [Fig. 4F]. The histology varied from crowded primitive appearing cells with hyperchromatic nuclei [Fig. 4A–B], to glial areas with a greater degree of cytoplasm [Fig. 4C], and less prevalent spindled areas [Fig. 4D]. This histology and staining pattern is similar to that described by Keck et al. [8], which reported desmin staining in 9/12 tumors ranging from rare positive cells to diffuse strong positivity. The *PLAGL2* amplified solid cellular tumor similarly showed desmin staining in a larger subset of neoplastic cells [Fig. 4I], with a greater degree of desmin positivity than for either GFAP or synaptophysin. The *PLAGL2* histology showed a vaguely perivascular arrangement of smaller embryonal appearing cells with hyperchromatic nuclei [Fig. 4G–H]. A similar loose perivascular/pseudopapillary architecture of primitive cells was also seen within areas of the *PLAGL1* amplified case [Fig. 4A]. Mitotic activity was elevated within primitive areas in both PLAG amplified cases, with 16 or 19 mitoses per 10 high power fields respectively.

### Molecular features

For cases with *EWSR1-PLAGL1* fusion, in 5/6 cases *EWSR1* (NM_05243.4) exon 8 was fused to *PLAGL1* (NM_001080951.2) exon 8, with one case having fusion of *EWSR1* (NM_05243.4) exon 7 to *PLAGL1* (NM_001080951.2) exon 8. Copy number changes showed focal alterations on chromosome 6q and 22q, near the locations of the *PLAGL1* (6q24.2) and *EWSR1* (22q12.2) genes. One *EWSR1-PLAGL1* case was found to have a *SMARCB1* truncating pathogenic mutation (p.R53* at 34% allelic frequency), which corresponded to the subclonal loss of INI1 observed by immunohistochemistry. Despite the presence of p53 nuclear positivity in the vast majority of neoplastic cells in case #1, a corresponding *TP53* mutation was not found. In cases with PLAG-family amplification, RNA sequencing demonstrated increased RNA expression of *PLAGL1* and *PLAGL2* respectively. The molecular findings for all cases, including methylation profiling calibrated score results, are summarized in Table 2.

### Clinical outcomes

Clinical follow-up was available for 5/6 patients with *PLAGL1* fusion (range: 11 months to 10 years) with various treatment modalities and extent of resection [Table 1]. Three patients developed recurrence, one at 7 months following treatment per ACNS0334 with intracranial multifocal disease and death at 11 months, one patient with two recurrences at 1.6 years and 9 years after chemotherapy, and one patient with residual/recurrent disease at 6 months and local rapid recurrence at 1.2 years. Outcome data for the *PLAGL2* amplified case showed no evidence of disease at 4.8 years, and available outcome data for the *PLAGL1* amplified case showed no definitive residual disease at 7 months.

## Discussion

It is likely that prior to clinical availability of RNA sequencing and methylation profiling, many cases with undetected *EWSR1-PLAGL1* fusion were histologically diagnosed as ependymoma, while some cases may have been considered a type of anaplastic ganglioglioma or high-grade neuroepithelial tumor. Given the limited number of molecularly confirmed cases with outcome data currently reported, it is yet unclear if future classification will consider supratentorial neuroepithelial tumors with ependymal features and *EWSR1-PLAGL1* rearrangement as a distinctive type of supratentorial ependymoma, or an entirely new entity. Interestingly, a mouse brain tumor model studying *ZFTA-RELA* fusion found that the ZFTA-RELA fusion protein product contains a C2H2 zinc finger domain similar to PLAGL1, and binds to genome sites enriched with PLAGL family transcription factor motifs [2], potentially recognizing a similarity in mechanistic transcriptional regulation between *ZFTA-RELA* fusion ependymomas and ependymal-like tumors with *PLAGL1* rearrangement. Gene expression analysis by Sievers et al. [15] between *ZFTA-RELA* fused ependymomas and the supratentorial neuroepithelial tumor *PLAGL1* fusion methylation group did demonstrate segregation of tumor samples. The weighting of different parameters such as methylation or mutational profiles with histology, imaging, immunostaining, and clinical course in determining how to classify tumors as distinctive, similar, or separate entities is an evolving area in tumor classification theory. While *PLAGL1* rearranged cases often have ependymal-like histology and similarly lack OLIG2/SOX10 immunostaining, the infrequent EMA paranuclear staining and other differences could be interpreted as distinctive from true ependymal lineage. Whether *EWSR1-PLAGL1* rearranged cases represent a novel molecular type of ependymoma or a distinctive neuroepithelial entity with histologic overlap warrants further evaluation to clarify this relationship, particularly for clinical therapeutic implications.

As there is variation among ependymal-like cases for proliferation rate and presence of necrosis, without clear associations for clinical outcome within a potentially molecularly defined entity, general characterization of *PLAGL1* rearranged neuroepithelial tumors as low-grade or high grade may yet be premature. Molecularly defined ependymomas similarly lack current meaningful outcome data for a WHO grade designation, supplementing a controversial history regarding ependymoma histologic grading [6, 7]. In *PLAGL1* rearranged cases with a combination of increased cellularity, elevated Ki-67, elevated mitotic index, and areas of necrosis the histologic features would support a higher-grade designation for therapeutic purposes. In this small series, the one patient with extended follow-up duration of 10 years had recurrent disease with a relatively indolent course given the length of time on clinical observation. In contrast, one patient died 11 months after initial resection with multifocal intracranial disease; however, this tumor additionally harbored a subclonal *SMARCB1* mutation and clusters of embryonal cells making it histologically and molecularly distinctive from the other cases. Other examples of AT/RT arising from glial or glioneuronal entities have often shown poor outcomes [5, 9, 11]. These differences emphasize the importance of reporting PLAG-family altered neuroepithelial tumors with their associated clinical and histopathologic correlates including treatment and outcome. Treatment decisions for rare and emerging entities are often made on a case-by-case basis with discussions at multidisciplinary brain tumor conferences and consideration of published literature or clinical trial availability. Although case numbers and length of follow-up were too limited in this series for correlation of outcome with patient age, mitotic indices, or degree of ganglionic differentiation, the worst prognosis among the *PLAGL1* fusion cases was associated with development of INI1 inactivation.

A feature observed in a subset of supratentorial neuroepithelial tumors with *EWSR1-PLAGL1* rearrangement is the presence of concurrent ependymal and ganglionic differentiation. Though not present in all cases and unlikely to be entity specific, the combined presence of ependymal and ganglionic features may raise consideration for a Supratentorial neuroepithelial tumor with *EWSR1-PLAGL1* fusion, and prompt initiation of appropriate testing such as RNA sequencing and methylation profiling. While these cases expand their presence within the literature, a descriptive diagnosis may be appropriate such as Supratentorial neuroepithelial tumor with ependymal and ganglionic features, *EWSR1-PLAGL1* rearranged. Additionally, given the poor outcome encountered in the *EWSR1-PLAGL1* case that acquired *SMARCB1* mutation, INI1 staining should be considered in *PLAGL1* fusion cases. It is important to recognize supratentorial neuroepithelial tumors with *EWSR1-PLAGL1* fusion as a tumor type in which acquired inactivation of *SMARCB1* and development of AT/RT features may occur and lead to clinical progression, similar to previously reported examples involving pleomorphic xanthoastrocytoma, ganglioglioma, and ependymoma [3, 5, 9, 11].

For *PLAGL1/PLAGL2* amplified cases the degree of desmin positivity was a distinctive feature in the two cases of this series, such that when present desmin positivity within a primitive appearing neoplasm could help to raise consideration for the diagnosis. Continued compilation of associated clinical data will be critical for understanding emerging entities with PLAG-family genetic alterations, to recognize their full spectrum of appearance and clinical behavior.

### Supplementary Information

Supplementary Material 1. Additional molecular testing methods.

Supplementary Material 2. (Supplementary Table 1) Detailed imaging characteristics of neuroepithelial tumors withPLAG-family genetic alterations.

## Data Availability

Supporting data available from the corresponding author upon reasonable request.
