# Supplementary material for: Concurrent ependymal and ganglionic differentiation in a subset of supratentorial neuroepithelial tumors with EWSR1-PLAGL1 rearrangement
Source: Acta Neuropathol Commun. 2024 Sep 3;12:143. doi: 10.1186/s40478-024-01809-9 (PMC11370057; doi:10.1186/s40478-024-01809-9)
Supplement: Supplementary file 1 — Supplementary Material 1. Additional molecular testing methods. [file 40478_2024_1809_MOESM1_ESM.docx]

**Supplementary File 1.** Additional molecular testing methods.

**Combined WGS/WES/RNA sequencing**

DNA extracted from the patient’s tumor specimen and germline specimen was used to construct libraries for WGS using Illumina TruSeq DNA LT PCR-Free Sample Kit and for WES using the Illumina TruSeq Exome Library Prep Kit. RNA was extracted from the patient’s tumor sample and the Illumina TruSeq Stranded Total RNA LT Kit was used to generate libraries for RNA-Seq. All libraries were sequenced using a paired end 2 x 125 bp cycle protocol and SBS technology on Illumina NovaSeq and/or NextSeq Instruments. Data from WGS, WES and RNA-Seq were analyzed independently to validate the presence of lesions in two or more platforms. A combined analysis was also performed to provide higher coverage particularly in cases with tumor heterogeneity, low tumor purity and/or for regions of the genome that are difficult to sequence due to high guanine cytosine content. Sequence information is aligned against a human reference sequence (Hg19) utilizing a custom bioinformatics pipeline. Variants found in the tumor were compared to the matched germline to eliminate private benign polymorphisms. Data analysis was performed using publicly available software, publicly available databases, proprietary databases commercially available, and databases/software developed at St. Jude Children’s Research Hospital. Tumor copy number variants, structural variants, fusion transcripts, single nucleotide variants, and small insertions and deletions (indels) classified as pathogenic or likely pathogenic are reported. Tumor variants categorized as of uncertain clinical significance, benign or likely benign are not reported. The following 115 genes are evaluated for germline variants in this assay: *AIP, ALK, AMER1, ANKRD26, APC, ATM, AXIN2, BAP1, BARD1, BLM, BMPR1A, BRAF, BRCA1, BRCA2, BRIP1, BUB1B, CBL, CDC73, CDH1, CDK4, CDKN1B, CDKN1C, CDKN2A, CEBPA, CHEK2, CTR9, DDX41, DICER1, DKC1, ELP1, EPCAM, ERCC4, ETV6, EXT1, EXT2, FANCA, FANCC, FANCG, FH, FLCN, GATA2, GPR161, GREM1, HRAS, IKZF1, KIF1B, KIT, KRAS, LZTR1, MAP2K1, MAP2K2, MAX, MEN1, MET, MITF, MLH1, MSH2, MSH3, MSH6, MUTYH, NBN, NF1, NF2, NRAS, NTHL1, PALB2, PAX5, PDGFRA, PHOX2B, PMS2, POLD1, POLE, POT1, PRKAR1A, PTCH1, PTCH2, PTEN, PTPN11, RAD51C, RAD51D, RAF1, RB1, RECQL4, REST, RET, RIT1, RRAS, RUNX1, SAMD9, SAMD9L, SDHA, SDHAF2, SDHB, SDHC, SDHD, SH2D1A, SHOC2, SMAD4, SMARCA4, SMARCB1, SMARCE1, SOS1, SOS2, SPRED1, STK11, SUFU, TERC, TERT, TMEM127, TP53, TRIM28, TSC1, TSC2, VHL, WT1*.

**RNA sequencing**

RNA was extracted from the patient’s tumor sample, and the Illumina TruSeq Stranded Total RNA LT Kit was used to generate libraries for RNA sequencing (RNA-Seq). All libraries were sequenced using a paired end 2 x 125 bp cycle protocol and SBS technology on Illumina NovaSeq and/or NextSeq Instruments. Data from RNA-Seq was analyzed to detect oncogenic gene fusions. RNA sequencing data was aligned against a human reference sequence (hg19) utilizing a custom bioinformatics pipeline. Data analysis was performed using publicly available software, publicly available databases, proprietary databases that are commercially available, and databases/software developed at St. Jude Children’s Research Hospital. Gene fusions were selected for review based on these resources, scientific literature, and other predefined variant features.

**Methylation Profiling**

DNA was extracted from the patient’s tumor sample and bisulfite converted using the Zymo EZ DNA methylation kit (Zymo Research). For formalin-fixed, paraffin-embedded (FFPE) tissues, the Illumina Infinium HD FFPE DNA Restore kit and the Zymo ZR96 DNA Clean and Concentrator kit were used. Methylation and copy number profiling were carried out with the Infinium MethylationEPIC BeadChip array (850k array) (Illumina Inc.) on the Illumina iScan platform. This array quantitatively targets 862,927 CpG sites across the genome. Data from the methylation array was analyzed for tumor classification and calculation of calibrated family and class scores via a custom-designed neural network classifier (St. Jude MLPnet version 3.0 and later) on a St. Jude Children’s Research Hospital HIPAA compliant high-performance computing system, using methylation data from 81,920 probes. Copy number variation analysis was performed using conumee-pipe() in the AnyCN package version 1.0 and later, with the parameters normalized by the Functional Normalization (funnorm) method and batch effect correction for copy number.

**Targeted panel sequencing on St. Jude Pedi Panel v1.1**

DNA extracted from the patient’s tumor specimen was used to construct libraries using the Twist Library Preparation EF Kit 2.0 and target regions from selected 362 genes were enriched. All libraries were sequenced using a paired-end 2 x 151 bp cycle protocol and SBS technology on Illumina NextSeq Instruments. Sequence information was aligned against a human genome reference sequence (hg19). Data analysis was performed using publicly available software, publicly available databases, commercially available proprietary databases, and other databases/software developed at St. Jude Children’s Research Hospital. This assay will detect single nucleotide variants (SNVs) and insertion/deletion (indel) variants of less than 25bp. Only coding regions (exons +/- 2bp neighboring intronic regions) are evaluated. Copy number alterations and translocations will not be detected. Variants categorized as of uncertain clinical significance, benign or likely benign are not reported. Sensitivity for certain variant types and specific panel target regions, such as sequences with high guanine cytosine content, may be reduced. There is limited ability to accurately identify variants occurring in regions with high homology to other regions of the genome (e.g., paralogous genes and pseudogenes). Analysis of PMS2 exons 11-15 is excluded due to high pseudogene interference. The following 362 genes are evaluated in this assay: *ABL1, ABL2, ACIN1, ACTB, ACVR1, AFF1, AFF4, AKT1, AKT2, AKT3, ALK, AMER1, APC, ARAF, ARID1A, ARID1B, ARID2, ASXL1, ASXL2, ATF7IP, ATM, ATRX, BAP1, BAZ1A, BCL11B, BCL2, BCL6, BCL9, BCOR, BCORL1, BCR, BEND2, BRAF, BRCA1, BRCA2, BRPF1, BTG1, C11orf95, CBFA2T3, CBFB, CBL, CCL22, CCND1, CCND2, CCND3, CD19, CD200, CD22, CD33, CDC73, CDH1, CDK4, CDK6, CDKN1B, CDKN1C, CDKN2A, CDKN2B, CDKN2C, CEBPA, CHD4, CHD7, CHEK1, CHEK2, CIC, CNOT3, CREBBP, CRLF2, CSF1R, CSF3R, CTCF, CTNNB1, CUX1, CXorf67, CXXC5, DAXX, DAZAP1, DDX3X, DEK, DGCR8, DHX15, DICER1, DMBT1, DNM2, DNMT3A, DROSHA, DUX4, EBF1, EED, EGFR, ELF1, ELL, ELP1, EP300, EPOR, ERBB2, ERBB3, ERBB4, ERG, ETV6, EWSR1, EZH2, FBXO11, FBXW7, FGFR1, FGFR2, FGFR3, FGFR4, FLI1, FLT3, FMR1, FOXO1, FOXR2, FPGS, FUBP1, FUS, GATA1, GATA2, GATA3, GLI1, GLI2, GLIS2, GNA11, GNA13, GNAQ, GNAS, GNB1, GSE1, H3F3A, H3F3B, HDAC7, HIST1H3A, HIST1H3B, HIST1H3C, HLF, HNF1A, HNRNPUL1, HOXA10, HOXD13, HRAS, ID3, IDH1, IDH2, IGH, IKZF1, IKZF2, IKZF3, IL3RA, IL7R, INO80, IRF4, ITPKB, JAK1, JAK2, JAK3, KBTBD4, KDM5A, KDM6A, KDR, KIAA1549, KIT, KLF4, KMT2A, KMT2B, KMT2C, KMT2D, KMT2E, KRAS, LDB1, LEF1, LMO1, LMO2, LYL1, MAP2K1, MAP2K2, MAP2K4, MAP3K14, MAP3K4, MAP3K8, MAPK1, MAX, MDM2, MDM4, MED12, MEF2D, MEN1, MET, MGA, MITF, MLF1, MLH1, MLLT1, MLLT10, MLLT3, MLLT4, MN1, MPL, MS4A1, MSH2, MSH6, MTOR, MUTYH, MYB, MYBL1, MYC, MYCN, MYH11, MYOD1, NCOR1, NF1, NF2, NIPBL, NKX2-1, NONO, NOTCH1, NOTCH2, NPM1, NR3C1, NR3C2, NRAS, NSD1, NT5C2, NTRK1, NTRK2, NTRK3, NUP214, NUP98, NUTM1, NUTM2A, NUTM2B, OTX2, P2RY8, PALB2, PAX3, PAX5, PAX6, PAX7, PBX1, PDGFRA, PDGFRB, PDS5B, PHF23, PHF6, PHIP, PICALM, PIK3C2G, PIK3CA, PIK3CB, PIK3CD, PIK3CG, PIK3R1, PIK3R2, PLAG1, PMS2, POLD1, POLE, PPM1D, PRKAR1A, PRPS1, PRPS2, PTCH1, PTEN, PTPN11, PTPN2, PTPRD, QKI, RAC1, RAD21, RAD50, RAF1, RAG1, RAG2, RB1, RBM15, RCSD1, RELA, RET, RHOA, RIT1, ROS1, RPL10, RPL11, RPL22, RPL5, RUNX1, RUNX1T1, SAMD9, SAMD9L, SDHA, SDHB, SEPTIN2, SEPTIN5, SEPTIN6, SEPTIN9, SETBP1, SETD2, SF3B1, SH2B3, SHANK2, SHH, SIX1, SIX2, SMAD4, SMARCA2, SMARCA4, SMARCB1, SMC1A, SMC3, SMO, SOS1, SRSF2, SSBP2, STAG1, STAG2, STAT3, STAT5B, STAT6, STK11, SUFU, SUZ12, TACC1, TAF15, TAL1, TAL2, TBL1XR1, TBR1, TCF3, TCF4, TCF7, TERC, TERT, TET2, TFAP4, TLX1, TLX3, TNFAIP3, TP53, TPM3, TRAF7, TSC1, TSC2, U2AF1, U2AF2, UBA2, UBTF, USP2, USP7, USP9X, VHL, WAC, WHSC1, WT1, XBP1, XPO1, YAP1, ZBTB7A, ZC3HAV1, ZEB2, ZFP36L2, ZIC1, ZMYM3, ZMYM4, ZNF384.*
